# Long Noncoding RNA* PCAT6* Regulates Cell Proliferation and Migration in Human Esophageal Squamous Cell Carcinoma

**DOI:** 10.7150/jca.62671

**Published:** 2022-01-01

**Authors:** Weibing Wu, Haixing Wei, Shuo Hu, Qi Wang, Chang Zhang, Jing Xu, Zhicheng He, Zhihua Li, Xianglong Pan, Jing Cao, Yining Zhu, Liang Chen

**Affiliations:** 1 Department of Thoracic Surgery, The First Affiliated Hospital of Nanjing Medical University, Nanjing, Jiangsu Province, China.; 2 Department of Thoracic Surgery, The First Affiliated Hospital of Anhui Medical University, Hefei, Anhui Province, China.; 3 Department of Thoracic Surgery, The First People's Hospital of Lianyungang, Lianyungang, Jiangsu Province, China.; 4 State Key Laboratory of Reproductive Medicine, Nanjing Medical University, Nanjing, Jiangsu Province, China.

**Keywords:** LncRNA, *PCAT6*, cell proliferation, cell migration, ESCC.

## Abstract

Esophageal squamous cell carcinoma (ESCC) is the sixth most common cancer type in East Asian countries. Mounting evidences illustrated that long noncoding RNAs (lncRNAs) play important roles in a variety of human cancers, including ESCC. LncRNA *PCAT6* has been identified as a tumor promoter in multiple cancers. However, the roles and underlying mechanism of *PCAT6* in ESCC remain largely unclear. In the current study, we discovered that lncRNA *PCAT6*, which was aberrantly upregulated in ESCC tumor tissues, significantly promoted cell proliferation and migration in ESCC cell lines Eca-109 and Kyse-30 cells. Flow cytometry assays showed that *PCAT6* knockdown promoted the apoptosis of ESCC cells. Mechanistically, RNA-seq and Gene Ontology analyses indicated that *PCAT6* knockdown influenced the expression of genes that participated in cell proliferation and migration. Furthermore, real-time PCR and western blot assays validated that knockdown of *PCAT6* could increase the levels of *GDF15* and *DUSP4* in Eca-109 and Kyse-30 cells. In brief, our findings reveal that lncRNA *PCAT6* plays an oncogenic role in the progression of ESCC by inhibiting the expression of genes related to cell proliferation and migration.

## Introduction

Esophageal cancer is a common malignant disease of the digestive tract and one of the six most common malignant tumors in the world. There are two types of esophageal carcinoma, esophageal squamous cell carcinoma (ESCC) and esophageal adenocarcinoma (EAC). EAC is highly prevalent in western countries, while ESCC is the most primary subtype in China. In 2018, it was estimated that there were 307 thousand newly diagnosed ESCC cases and 283 thousand ESCC related deaths in China 1, 2. Although the diagnosis and treatment techniques of ESCC are developing rapidly, the prognosis is still poor. The 5 year overall survival of ESCC patients is less than 30% in most countries 3. Therefore, it is very important to further explore the pathogenesis of esophageal cancer to identify more crucial biomarkers and therapeutic targets.

In recent years, owing to the development of Encyclopedia of DNA Elements project, long non-coding RNA (lncRNA) has attracted more and more attention 4, 5. LncRNA is one of the RNA transcripts with approximately 200 to 100000 nucleotides in length 6, 7. With in-depth research, the biological functions of lncRNAs have been initially revealed. LncRNAs participate in a series of cellular progressions, such as cellular development, apoptosis, transcriptional regulation, intracellular material transport and chromosome remodeling, *etc.* 8-11. Moreover, the abnormal expression of lncRNAs has been established to play significant roles in regulating tumorigenesis including ESCC 12-19.

Prostate cancer associated transcript-6 (*PCAT6*), which has been reported in previous studies, was found upregulated in tumor tissues and could promote the tumor progression in multiple cancer types 20-23. For example, Wan* et al* observed that *PCAT6* was significantly elevated in lung tumor tissues and patients with a high expression of *PCAT6* showed poorer prognosis. Functionally, they discovered that knockdown of *PCAT6* significantly repressed the proliferation and invasion of lung cancer cells 20. Similarly, the expression of *PCAT6* was aberrantly elevated in colorectal cancer (CRC) tissues and knockdown of *PCAT6* attenuated CRC chemoresistance to 5‐FU 21. Nevertheless, the specific function of *PCAT6* in the development of ESCC is still unclear.

In this study, we found that *PCAT6* was significantly upregulated in ESCC tissues, and we confirmed that *PCAT6* could promote cell proliferation and migration by the loss and gain of function assays. Flow cytometry assays showed that *PCAT6* knockdown promoted the apoptosis of ESCC cells. In addition, RNA-seq and Gene Ontology (GO) analysis suggested that *PCAT6* associated genes were mainly participated in pathways such as cell proliferation and cell migration. Prior to this, the downstream global gene expression profile of *PCAT6* had not been studied, and we determined global gene expression profiling regulated by *PCAT6* for the first time. The real-time PCR and western blot assays further proved that knockdown of *PCAT6* could upregulate the expression of *GDF15* and *DUSP4*, both of which have been reported associated with cell proliferation. In conclusion, our study reveals that lncRNA *PCAT6* functioned as a tumor promoter in the progression of ESCC by regulating the expression of genes related to cell proliferation and migration.

## Materials and Methods

### Human samples and cell lines

Tissues used in this study were collected from 44 patients with ESCC underwent esophagectomy at the First Affiliated Hospital of Nanjing Medical University. All collected tissue specimens were immersed in RNA Later stabilization solution (Qiagen) and were immediately frozen in liquid nitrogen and conserved at - 80 °C until RNA extraction. This study was approved by the Research Ethics Committee of the First Affiliated Hospital of Nanjing Medical University, and written informed consent was acquired from all the patients.

ESCC cell lines (Eca-109, Kyse-30, TE-1, Kyse-70, Kyse-150) and human esophageal mucosal epithelial cell line (Het-1A) were purchased from Shanghai Cell Bank (Shanghai, China). These cells were cultivated in RPMI-1640 or DMEM medium (GIBCO‐BRL) with 10% fetal bovine serum (FBS), 100 U/ml penicillin and 100 mg/ml streptomycin. Cells were incubated at 37 °C 5% CO_2_.

### Transfection of cell lines

We transfected Eca-109 and Kyse-30 cell lines with the siRNAs targeting *PCAT6* or si-NC by utilizing Lipofectamine-2000 (Invitrogen) based on the protocol. The plasmid was transfected into Eca-109 and Kyse-30 cells with the X-tremeGENE™ HP DNA Transfection Reagent (Roche) following the manufacturer's protocol. After transfection for 48h, we harvested the cells for qRT-PCR to quantify the expression of related genes. All sequences of siRNAs used in this study were shown in **Supplementary Table S1**.

### RNA extraction, reverse transcription and qRT-PCR

Total RNA was isolated from ESCC cells using TRIzol reagent (Invitrogen, Carlsbad, CA) following the instructions. 1μg of RNA from each specimen was reverse transcribed into complementary DNA (cDNA) for qRT‐PCR by utilizing the Reverse Transcription Kit (Takara, Dalian, China). We used SYBR PCR Master Mix reagent kit (Takara, Dalian China) for qRT‐PCR assays based on the publisher's protocol. The result was standardized with the endogenous expression of GAPDH. All the relative primer sequences were presented in **Supplementary Table S1**.

### Cell proliferation assay

Cell proliferation assay was examined by using Cell Proliferation Reagent Kit I (MTT) (Sigma) following the standard procedures. After transfection for 24h, Eca-109 or Kyse-30 cell lines were placed in 96-well plates. We tested the cell viability every 24h based on the protocol. After cells transfection with si-*PCAT6* or si-NC for 24h, a certain amount of cell suspension was grown in six-well plates for colony formation experiments. They were cultured in the cell incubator with 5% CO_2_ at 37 °C for ten days, replacing the fresh RPMI-1640 medium every 5 days. Then cells were treated with methanol for 30 mins and dyed by using crystal violet for 15 mins. We counted the number of clones and compared the results. All experiments were performed three times independently.

### Ethynyldeoxyuridine (EdU) analysis

According to the instruction, we utilized an EdU labeling/detection kit (Ribobio, China) to valuate proliferating cells. Eca-109 or Kyse-30 cells were placed in 24-well plates at 3*10^^^4 cells per well. After transfection for 48h, the cell culture was replaced with 50 μM EdU labeling medium and they were still incubated for 4h in a cell incubator Then, the cells were fixed with 4% paraformaldehyde (pH 7.4) for 30 mins and then treated with 0.5% Triton X-100 for 15 mins at room temperature. Next, we stained the cells by using anti-EdU working solution for 15 mins. Finally, the cells were incubated with 300 μl Hoechst 33342 (5 μg/ml). The ratio of EdU-positive cells was calculated and compared. The assay was repeated three times independently.

### Cell migration assays

After transfection for 24 h, 6×10^4^ Eca-109 or 3×10^4^ Kyse-30 cells in medium containing 1% FBS were seeded in the upper chambers of inserts (Millipore, Billerica, USA). The medium including 10% FBS was added to the lower chamber. The cells were treated with methanol and 0.1% crystal violet 48 hours after incubation. Then we wiped away the cells that remained on the upper chamber with the cotton swab. The cell which had migrated through the membrane was evaluated through the inverted microscope. The assay was replaced in triplicate independently.

### Flow cytometric analysis of apoptosis

Following the instructions, ESCC cells were harvested 48 hours after transfection and performed Annexin V-FITC and Propidium iodide for 10 mins with the Annexin V-FITC Apoptosis Detection Kit (Vazyme BioTech, Nanjing, China). Next, the cells were conducted by flow cytometry (FACScan®; BD Biosciences) for analysis of apoptosis.

### RNA-seq bioinformatic analysis

After Eca-109 cell lines treated with si-*PCAT6*-1# or si-NC for 48 hours, total RNA was extracted and quantified separately. The concentration of each specimen was measured with using NanoDrop 2000. The Agilent2200 (Agilent, USA) was conducted for assessing the quality. The sequencing library of every RNA specimen was established with using Ion Proton Total RNA-Seq Kit v2 based on the protocol provided by manufacturer (Life technologies, USA).

### Gene ontology analysis

In order to explore the biological implications of representative profiles of the target gene of the differentially expressed mRNA in the assay, GO analysis was further performed by using GO annotations from three public-available databases, including NCBI, UniProt and the Gene Ontology.

### Western blot

ESCC cells transfected with the specific siRNAs were lysed by utilizing RIPA buffer supplemented with Proteinase inhibitor. Total protein was extracted and separated with 10% SDS-PAGE gel, then transferred to a 0.45 μm polyvinylidene fluoride (PVDF) membrane. Then the PVDF membrane was incubated with specific antibodies. The protein was visualized through the autoradiography. The bodies (anti-*GDF15* and anti-*DUSP4*) were purchased from Abcam.

### Statistical analysis

The expression of *PCAT6* in ESCC tumor tissues and adjacent normal tissues was obtained from TCGA database. Association between *PCAT6* expression and the prognosis of ESCC patients was evaluated using Log-rank test. Student's t test was adopted to examine the differences between NC cells and *PCAT6* knockdown cells. All statistical analyses were performed based on R software v3.6.0 or GraphPad Prism v.7.00. In this study, *P* values less than 0.05 were regarded as statistically significant.

## Results

### *PCAT6* is upregulated in ESCC tumor tissues

**Figure 1:**
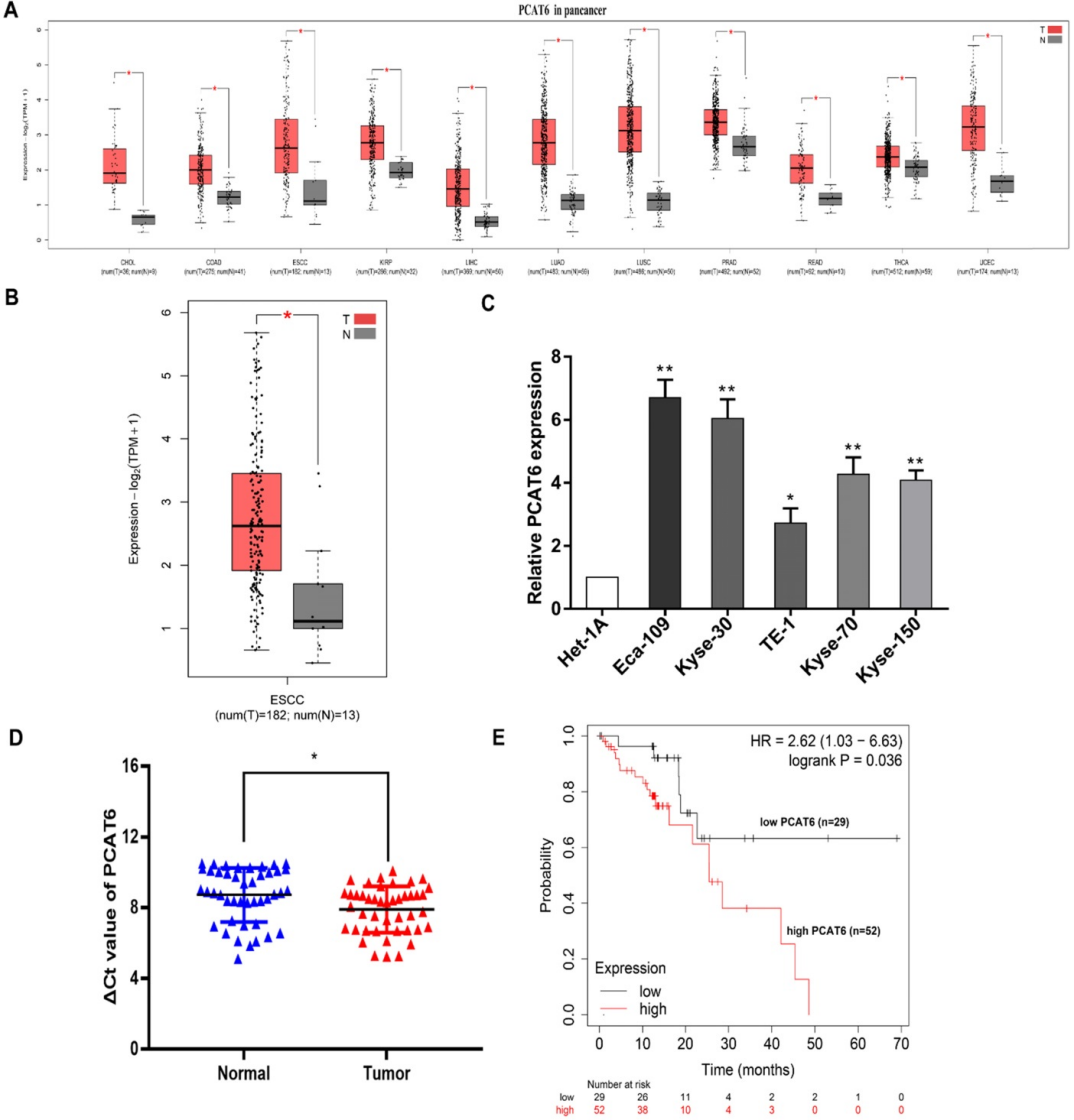
** Identification of the expression of *PCAT6* in ESCC. (A)** Data from TCGA database showed the relative expression of lncRNA *PCAT6* in several cancers and normal tissues.** (B)** The relative expression of *PCAT6* in in ESCC (n = 182) and nontumorous tissues (n = 13) from TCGA database. **(C)** *PCAT6* expression evaluated using qRT-PCR in ESCC cell lines (Eca-109, Kyse-30, TE-1, Kyse-70, Kyse-150) and normal esophageal mucosal epithelial cell line (Het-1A). **(D)** qRT-PCR analysis of the expression of *PCAT6* expression in 44 pairs of ESCC tissues and adjacent normal tissues collected from our center. The ΔCt value was determined by subtracting the GAPDH Ct value from the PCAT6 Ct value. A smaller ΔCt value indicates higher expression. **(E)** Kaplan-Meier survival plots revealed that higher *PCAT6* expression correlated with poor overall survival in ESCC patients. (*P < 0.05 and **P < 0.01)

To identify the clinical significance of *PCAT6*, we initially analyzed The Cancer Genome Atlas (TCGA) database (Figure 1A). Higher expression levels of *PCAT6* were identified in a variety of cancers through pan-cancer analysis. And differential expression of *PCAT6* was observed in the database which containing 182 ESCC tumor tissues and 13 normal tissues were included. From the results, we observed that* PCAT6* was significantly elevated in ESCC tumor tissues (Figure 1B). After that, we examined the expression of *PCAT6* in five ESCC cells and control cells. *PCAT6* was noticeably upregulated in ESCC cells than in control cells (Figure 1C). Eca-109 and Kyse-30 with higher levels of *PCAT6* were chosen for the following assays. To confirm the results, we collected 44 pairs of ESCC tissues and adjacent normal tissues from our center. qRT-PCR analysis demonstrated that PCAT6 was significantly upregulated in ESCC patients (Figure 1D). Further, we explored the relationship between *PCAT6* expression levels and prognosis of patients with ESCC by Kaplan-Meier Plotter analysis 24. As shown in **Figure 1E**, the results demonstrated that higher expression of *PCAT6* were correlated with significantly shorter overall survival. From the above, *PCAT6* may play a vital role in the tumorigenesis of ESCC.

### Knockdown of *PCAT6* inhibits ESCC cells proliferation and migration

**Figure 2:**
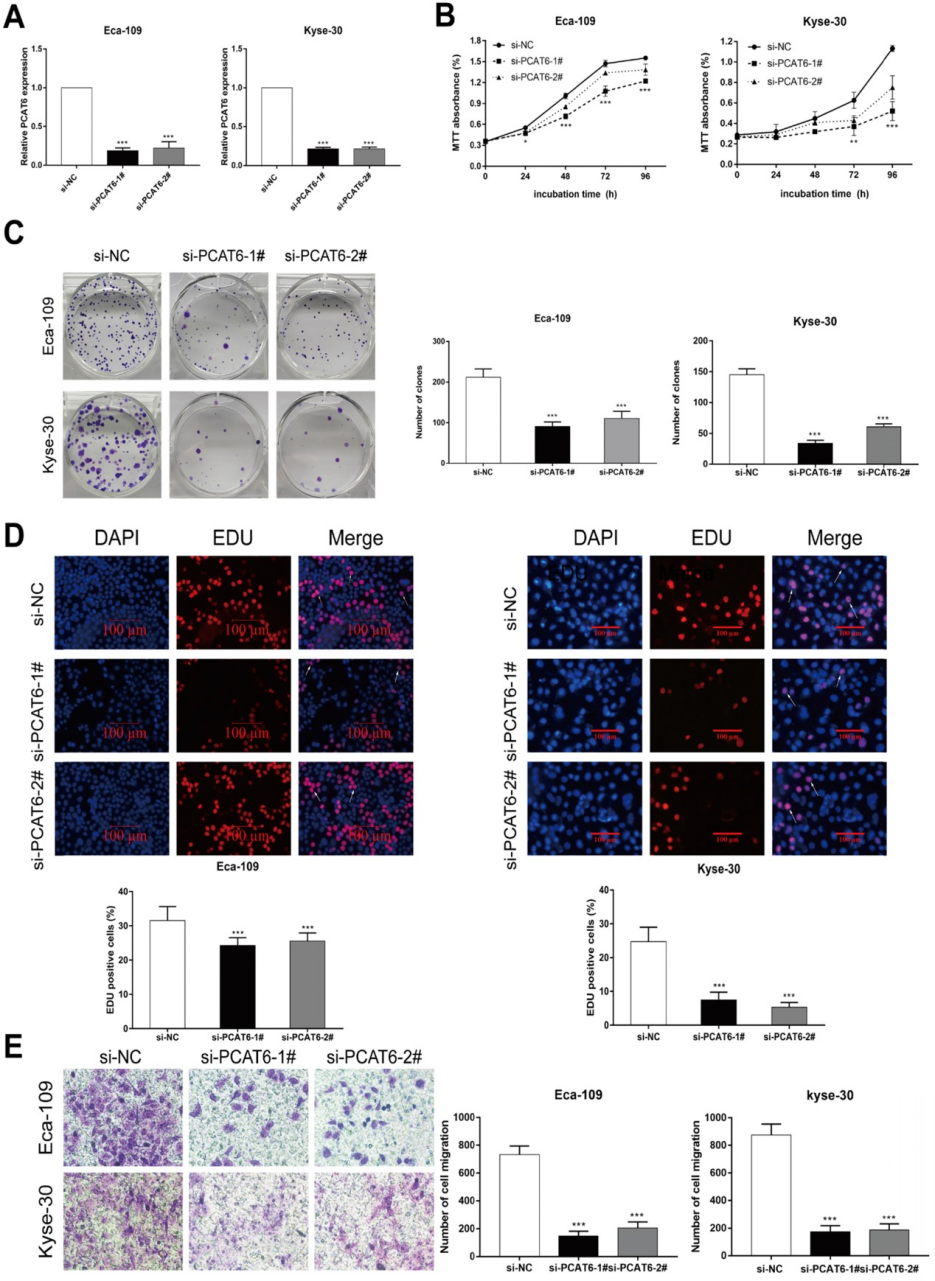
** The downregulation of *PCAT6* regulates ESCC cells proliferation and migration. (A)** The expression of *PCAT6* was tested using qRT-PCR after transfection with siRNAs in Eca-109 and Kyse-30 cells.** (B)** MTT assays were performed to confirm the viability of cell treated with *PCAT6* siRNAs. *PCAT6* knockdown significantly inhibited the viability of Eca-109 and Kyse-30 cells. **(C)** Colony formation assays exhibited that *PCAT6* knockdown significantly inhibited the proliferation of Eca-109 and Kyse-30 cells. **(D)** EDU staining assays were conducted to determine cells proliferation following knockdown of *PCAT6*. The arrow points to the positive cell. Scale bars = 100 μm. **(E)** The migration abilities of Eca-109 and Kyse-30 cells were examined by transwell assays after transfection, respectively. (Data are mean ± SD. **P < 0.01).

To investigate the biological role of *PCAT6* in ESCC tumorigenesis, we first knocked down the expression level of *PCAT6* using siRNAs targeting *PCAT6*. As shown in **Figure 2A**, the expression of *PCAT6* in Eca-109 and Kyse-30 cells was significantly decreased after transfection with *PCAT6* siRNAs. MTT assays indicated that cell viability was significantly inhibited when *PCAT6* levels were decreased in Eca-109 and Kyse-30 cell lines (**Figure 2B,** *P* < 0.001). Similarly, the colony formation experiment showed that the growth ability of ESCC cells with *PCAT6* knockdown was inhibited in both Eca-109 and Kyse-30 cells (**Figure 2C,** *P* < 0.001). Moreover, EdU staining assays also revealed that *PCAT6* had a significant influence on the proliferation of ESCC cells (**Figure 2D,** *P* < 0.001). In addition, down-regulation of *PCAT6* significantly suppressed cell migration ability in both Eca-109 and Kyse-30 cells (**Figure 2E,*** P* < 0.001). Our findings indicated that *PCAT6* could promote the proliferation and migration of ESCC cells.

### *PCAT6* promotes cell proliferation and migration in ESCC

**Figure 3:**
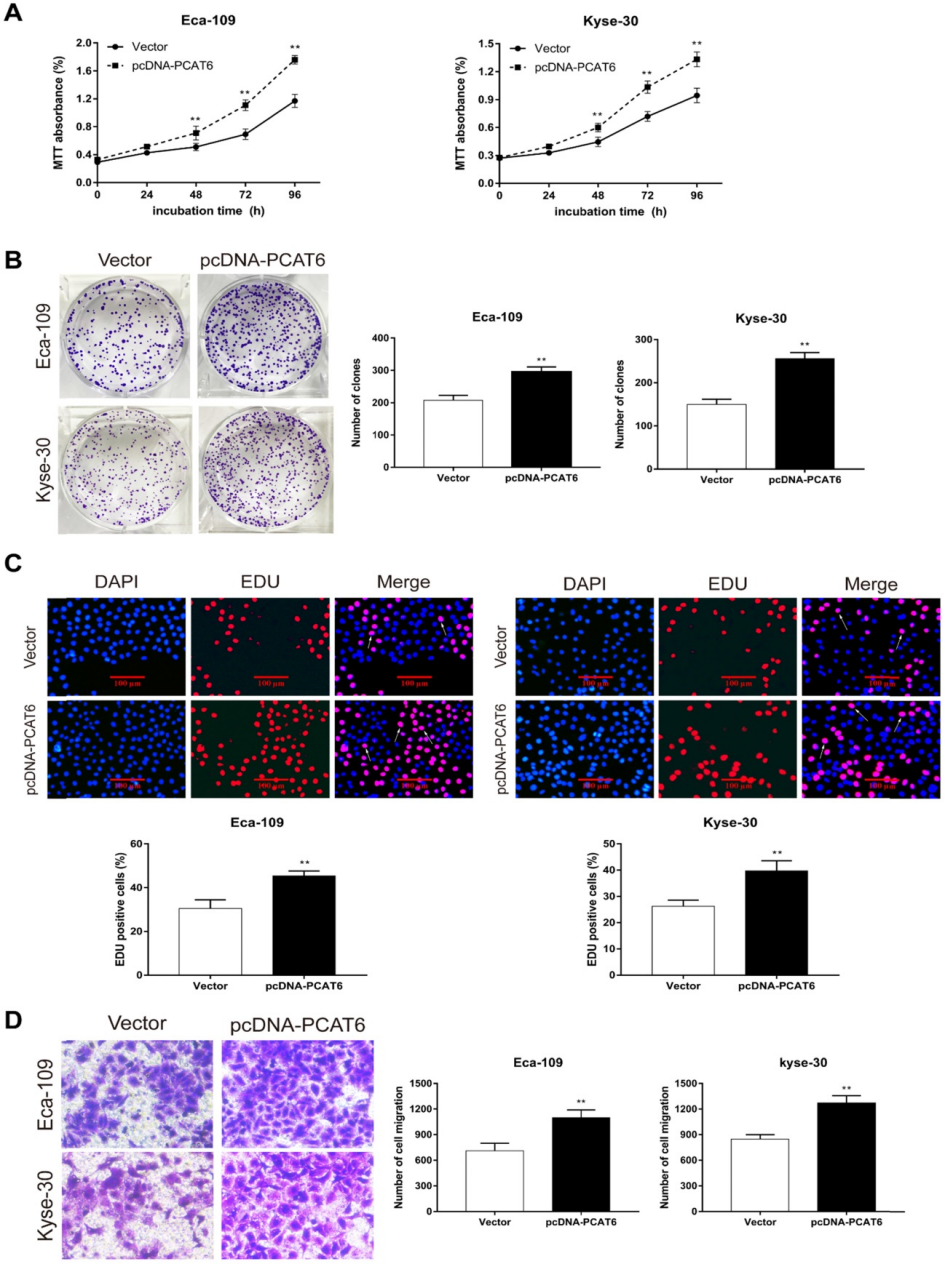
** Effect of overexpressed *PCAT6* on cell proliferation and migration in ESCC. (A)** MTT analysis of Eca-109 and Kyse-30 cells transfected with an overexpression plasmid of *PCAT6*. **(B)** Colony formation assays were conducted to determine cell proliferation of Eca-109 and Kyse-30 cells treated with the overexpression plasmid of *PCAT6*. **(C)** Edu assays of Eca-109 and Kyse-30 cells transfected with an overexpression plasmid of *PCAT6*. The arrow points to the positive cell. Scale bars = 100 μm. **(D)** Transwell assays were performed to detect the cell migration ability of Eca-109 and Kyse-30 cells following overexpressed *PCAT6*. (Data are mean ± SD. **P < 0.01).

To conform the oncogenic role of *PCAT6* investigate the effect of overexpression of *PCAT6* on ESCC cells proliferation and migration, we conducted corresponding cell functional assays. As shown in **Figure 3A**, MTT analysis revealed that *PCAT6* significantly increased cell activity in Eca-109 and Kyse-30 cells (*P* < 0.01). Then, colony formation assays were also performed, and the results indicated that the clonogenic ability of ESCC cells with *PCAT6* upregulation was improved (**Figure 3B**, *P* < 0.01). Furthermore, Edu staining experiments also showed that overexpression of *PCAT6* promoted cell proliferation (**Figure 3C**, *P* < 0.01). Additionally, transwell experiments demonstrated that overexpressed *PCAT6* significantly increased cell migration ability in ESCC cells (**Figure 3D**, *P* < 0.01). Together, these results demonstrate that *PCAT6* exerts an oncogenic role in ESCC through affecting cell proliferation and migration.

### *PCAT6* knockdown promotes ESCC cells apoptosis

**Figure 4:**
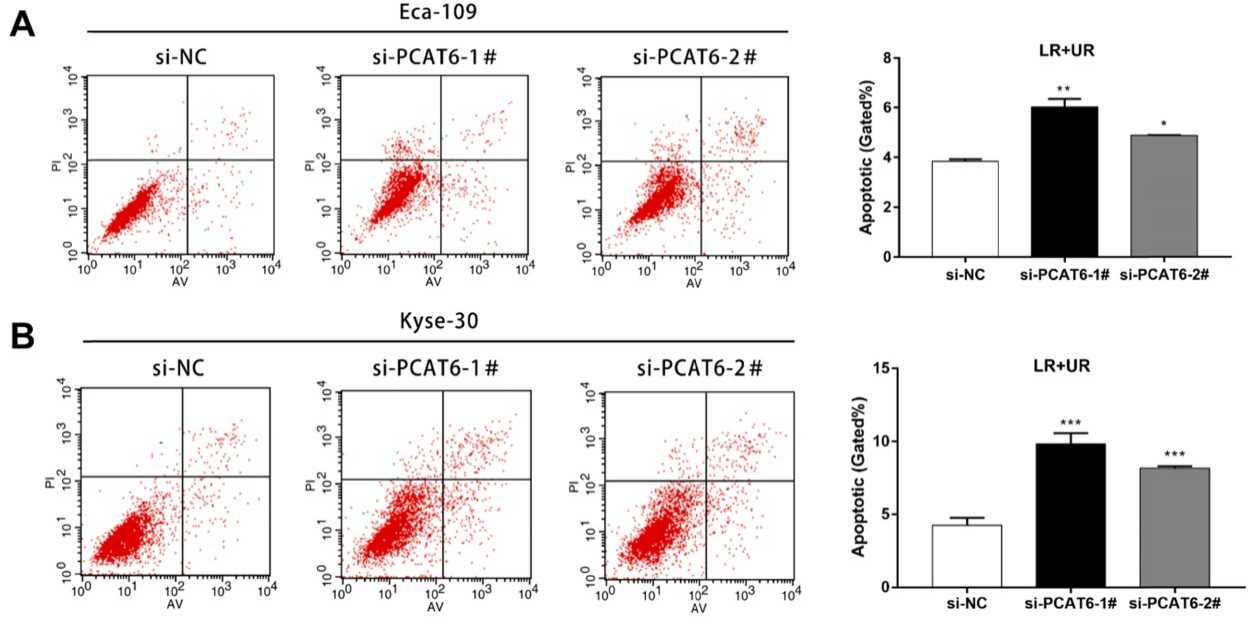
** Effect of* PCAT6* on cell apoptosis in ESCC cells.** Flow cytometry assays were performed to detected the cell apoptosis in **(A)** Eca-109 and **(B)** Kyse-30 cells. *PCAT6* knockdown resulted in increased cell apoptotic rate. LR, early apoptotic cells. UR, terminal apoptotic cells. (Data are mean ± SD. *P < 0.05, **P < 0.01 and ***P < 0.001).

Furthermore, flow cytometry was applied to confirm whether knockdown of *PCAT6* affected ESCC cells apoptosis. As presented in **Figure 4A**, the rate of both early apoptotic and late apoptotic cells significantly increased when *PCAT6* was down-regulated in Eca-109 cells compared to NC cells (*P* < 0.001). Consistently, *PCAT6* inhibition in Kyse-30 cells resulted in a similar inducement of apoptotic cells (**Figure 4B,** *P* < 0.001). Accordingly, our results demonstrated that *PCAT6* facilitates proliferation ability by regulating apoptosis in ESCC cells.

### The global gene expression profile regulated by *PCAT6* in ESCC Cells

**Figure 5:**
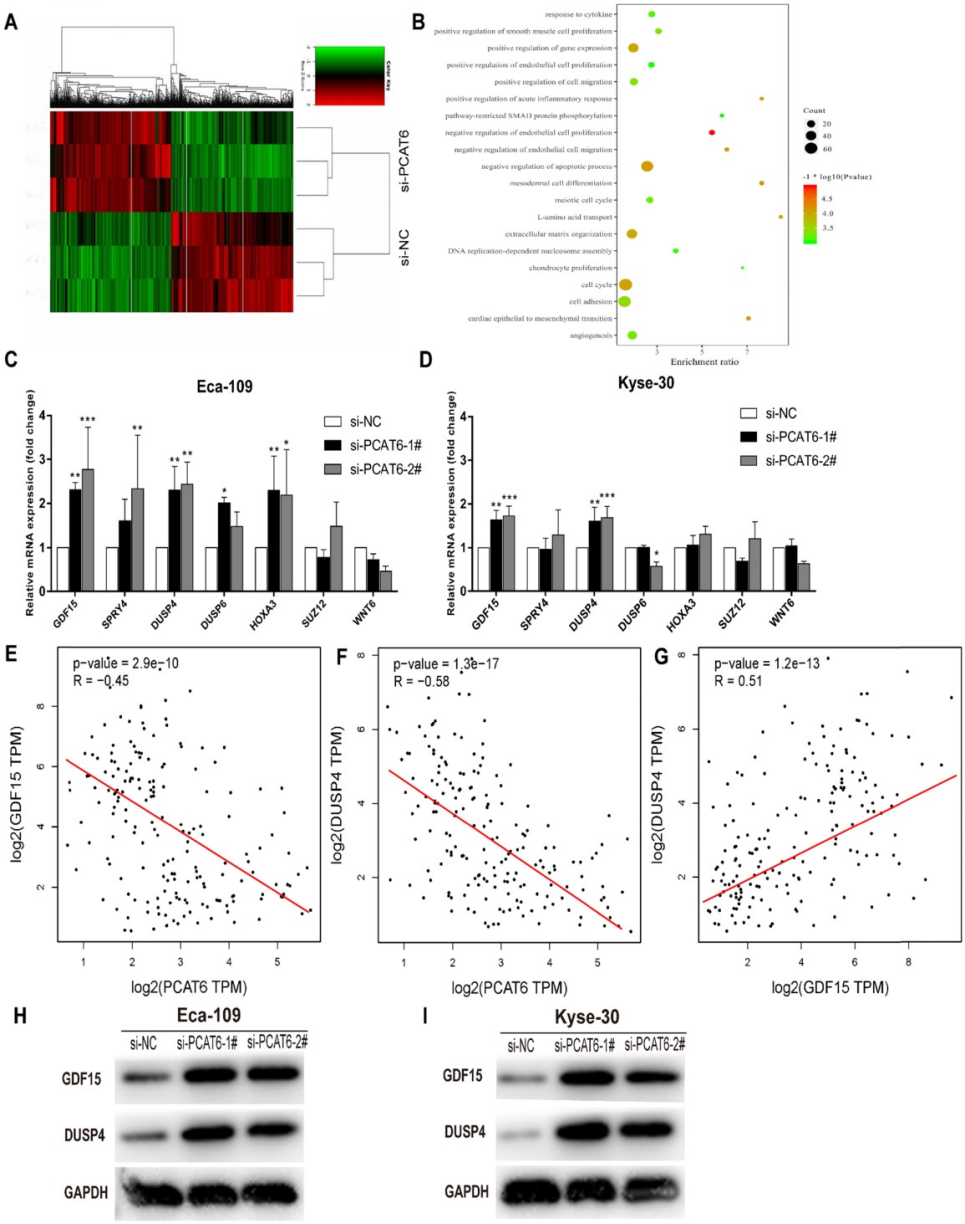
** The global gene expression profile regulated by *PCAT6* and underlying target genes. (A)** Mean-centered, hierarchical clustering of 1,278 gene transcripts altered (≥ 2-fold change) in the control and si-*PCAT6*-1#-treated Eca-109 cells, with three repeats. Red represents the high expression value, while green represents the low. **(B)** Gene ontology analysis for all genes with altered expressions. qRT-PCR was used to determine the changes of the altered genes expression which related to the cell proliferation after *PCAT6* knockdown in **(C)** Eca-109 and **(D)** Kyse-30 cells. **(E and F)** The respective negative correlations were found between the expression levels of *PCAT6* and *GDF15*, *DUSP4* in TCGA ESCC samples.** (G)** Correlation between *GDF15* and *DUSP4* mRNA from TCGA database. Western blot assays were conducted to verify the change in the protein levels of related genes following knockdown of *PCAT6* in **(H)** Eca-109 and **(I)** Kyse-30 cells. (*P < 0.05, **P < 0.01 and ***P < 0.001).

Although *PCAT6* has been reported to promote cell proliferation in a variety of cancers, the global gene expression profile regulated by *PCAT6* has not been clarified. In order to probe the molecular mechanisms underlying the increased proliferation of *PCAT6* in ESCC, RNA-Seq of *PCAT6* knockdown Eca-109 cells and control cells was carried out. As a consequence, the transcript levels of 775 mRNAs displayed ≤ 2-fold down-regulation in abundance in Eca-109 cells following *PCAT6* knockdown, while 503 protein-coding genes showed ≥ 2-fold up-regulation (**Figure 5A**; data are available in **Supplementary Table S2**). Furthermore, GO enrichment analysis demonstrated that these genes were mainly enriched in cell proliferation, cell migration pathways (**Figure 5B**).

As described above, *PCAT6* might participate in pathways associated with cell growth, migration and apoptosis in ESCC. As expected, we found that many well-known genes that associated with cell proliferation and migration (e.g. *WNT6*, *DUSP6*, *HOXA3*, *SUZ12*, *DUSP4*, *SPRY4*, *GDF15*, *et al.*) were involved in these pathways. To determine the potential target genes of *PCAT6*, we used qRT-PCR to verify the expression changes of the 7 genes. *GDF15*, *SPRY4*, *DUSP4* and *HOXA3* were observed upregulated in Eca-109 cells while* GDF15* and *DUSP4* upregulated in Kyse-30 cells with the suppression of *PCAT6* (**Figures 5C and D**). The consistent changes of *GDF15* and *DUSP4* levels in Eca-109 and Kyse-30 cells implies the potential regulatory roles of *PCAT6.* Previous studies have reported that *GDF15* had anti-tumorigenic and pro-apoptotic activity, which was first identified in colorectal cancer 25. In addition, overexpression of *DUSP4* in glioblastoma cells could result in a significant decrease of cellular proliferation ability specifically via dephosphorylating and inactivating MAPKs 26. Moreover, we analyzed the respective relationships between the expression levels of *PCAT6* and *GDF15*, *DUSP4* in TCGA ESCC samples. As shown in **Figure 5E and F**, the expression of *PCAT6* was negatively associated with the expression of *GDF15* and* DUSP4* in ESCC. Further , through exploring the correlation between GDF15 and DUSP4 in ESCC samples from TCGA, we found that there is a positive relationship between GDF15 and DUSP4 (**Figure 5G**).Western blot assays were further carried out to verify the expression changes of *GDF15* and *DUSP4* following *PCAT6* knockdown in Eca-109 and Kyse-30 cells. The protein levels were reduced by *PCAT6* knockdown (**Figure 5H and I**). These evidences suggested that the dysregulated genes *GDF15* and *DUSP4* might be the underlying downstream mediators of *PCAT6*.

## Discussion

Increasing evidences showed that lncRNAs played vital roles in cell growth and human disease development, especially in cancer 27, 28. The biological functions and roles of lncRNA *PCAT6* in multiple cancers have been revealed in previous studies. For example, high *PCAT6* expression was significantly associated with TNM stage and indicated poor overall survival of patients with lung cancer 20. Shi *et al*. found that *PCAT6* exerted the oncogenic activity by suppressing LATS2 through binding to the epigenetic repressor EZH2 in non-small-cell lung cancer 29. Moreover, *PCAT6* promoted cell proliferation and suppressed cell apoptosis via sponging miR-143-3p to upregulate PRDX5 in gastrointestinal stromal tumor 30. However, the possible role of *PCAT6* in ESCC is still unclear.

In the current study, we found that *PCAT6* was significantly upregulated in ESCC tumor tissues, suggesting that *PCAT6* might function as a tumor promoter in the progression of ESCC. As expected, knockdown of *PCAT6* in Eca-109 and Kyse-30 cells significantly inhibited the proliferation of ESCC cells. Consistently, the migration ability of ESCC cells was apparently suppressed in *PCAT6* knockdown cells compared to control cells. Furthermore, the cell apoptosis assay showed that down-regulation of *PCAT6* significantly promoted the apoptosis of ESCC cells in both Eca-109 and Kyse-30 cells. On the contrary, overexpression of *PCAT6* significantly promoted ESCC cells proliferation through the MTT, clone formation and Edu analysis. And the ability of ESCC cells migration was improved following overexpressed *PCAT6*. Taken together, all these findings revealed that *PCAT6* played an oncogenic role in ESCC tumorigenesis.

To further investigate the potential mechanisms of *PCAT6* in ESCC, RNA transcriptome sequencing was conducted. We obtained the downstream global gene expression profile of *PCAT6* in Eca-109 cells. GO enrichment analysis indicated that *PCAT6* associated genes were enriched in pathways related to cell proliferation and migration, which suggested that *PCAT6* could regulate the expression of genes involved in cell proliferation or migration. Further, the results of qRT-PCR and western blot proved that inhibition of *PCAT6* significantly increased the expression of *GDF15* and *DUSP4*, suggesting *GDF15* and *DUSP4* could be the target genes of *PCAT6*. Except for GDF15 and DUSP4, *HOXA3* was also observed to be upregulated in Eca-109 cells, it indicates that *PCAT6* might be involved in other regulatory pathways which needs further exploration.

*GDF15*, belonging to the TGF-β superfamily, has been reported to serve as a tumor suppressor in several cancers, including esophageal carcinoma 31-34. Lu X *et al* observed that *GDF15* was apparently downregulated in non-small-cell lung cancer tissues. Functional experiments indicated that overexpression of *GDF15* significantly reduced cell proliferation and induced apoptosis 33. In our study, *GDF15* was upregulated along with the knockdown of *PCAT6*. It indicated that *PCAT6* may promotes development of ESCC via suppressing *GDF15*. With regard to *DUSP4*, previous research reported that downregulation of *DUSP4* enhanced cell proliferation in colorectal carcinomas (CRC). Recovery of *DUSP4* led to inactivation of ERKs, inhibiting the proliferation and invasiveness of CRC cells 35. Zhang *et al* had illuminated that down-regulation of *DUSP4* expression in gastric cancer patients was associated with clinicopathological features, including tumor size and distant metastasis. Overexpression of *DUSP4* effectively suppressed the proliferation of gastric cancer cells and induced apoptosis 36. However, the roles and functions of *DUSP4* in ESCC have not been revealed in previous studies. Our results confirmed that *DUSP4* was upregulated following *PCAT6* knockdown. *PCAT6* may enhance ESCC cell proliferation by down-regulating *DUSP4*. Highlight these evidences, we speculated that *PCAT6* could promote the progression of ESCC by regulating the expression of *GDF15* and *DUSP4*. In addition, positive relationship between *GDF15* and *DUSP4* indicated their synergy effect to cell proliferation and migration.

To conclude, our findings revealed that lncRNA *PCAT6* was aberrantly elevated in ESCC tissues, and elucidated that *PCAT6* played an oncogenic role in ESCC cells. We also determined the global gene expression profile regulated by *PCAT6* by RNA-seq assays, and *GDF15* and *DUSP4* were identified as potential targets of *PCAT6*. Though further explorations were needed, our results provide a better understanding of the role of *PCAT6* in tumor progression and a potential therapeutic target and prognostic predictor against this ESCC.

## Supplementary Material

Supplementary table 1.

Supplementary table 2.

## Abbreviations

lncRNAs: long non-coding RNAs
ESCC: esophageal squamous cell carcinoma
EAC: esophageal adenocarcinoma
*PCAT6*: Prostate cancer associated transcript-6
GO: Gene Ontology
